# Molecular detection of relapsing fever *Borrelia puertoricensis* in migratory Mexican free-tailed bats

**DOI:** 10.1128/msphere.00085-26

**Published:** 2026-06-02

**Authors:** Daniel J. Becker, Kristin E. Dyer, Beckett L. Olbrys, Mackenzie G. Hightower, Meagan Allira, Bret Demory, Lauren R. Lock, Kiersten N. Taylor, Nakib N. Bhata, Selena M. Hernandez, Paul A. Lawson, Noha H. Youssef, Samuel L. Miller, Mostafa S. Elshahed, Taylor B. Verrett, Kerry L. Clark

**Affiliations:** 1 School of Biological Sciences, The University of Oklahoma 6187, Norman, Oklahoma, USA; 2 Department of Public Health, University of North Florida 4127, Jacksonville, Florida, USA; 3 Department of Microbiology and Molecular Genetics, Oklahoma State University 539937, Stillwater, Oklahoma, USA; Medical College of Wisconsin, Milwaukee, Wisconsin, USA

**Keywords:** *Tadarida brasiliensis*, migration, borreliae, tick-borne disease, zoonosis

## Abstract

Bats have been increasingly recognized to host relapsing fever borreliae as well as borreliae that form novel clades adjacent to the Lyme borreliosis group. However, the genetic diversity and zoonotic potential of bat-borne borreliae remain poorly understood, in part because most work to date has focused on bats in the tropics. Fewer bat-borne *Borrelia* surveys have been conducted in temperate zones, where many bats undertake seasonal migrations that may facilitate pathogen dispersal. We surveyed blood from nearly 400 Mexican free-tailed bats (*Tadarida brasiliensis*) during their seasonal occupancy in Oklahoma, USA, during 2022 and 2023, for *Borrelia* spp. Targeted PCR of the 16S rRNA and *flaB* genes revealed high nucleotide identity to *Borrelia puertoricensis*, and shotgun metagenomics further demonstrated high amino acid identity to strains isolated from argasid ticks and human blood. This represents the first detection of *Borrelia puertoricensis* in bats and only the second detection within wild vertebrate hosts. Infection prevalence was low but comparable to that of other borreliae in bats. Our findings suggest that Mexican free-tailed bats may contribute to the dispersal of this emerging tick-borne bacterial pathogen in North America.

**IMPORTANCE:**

Bacteria in the genus *Borrelia* are primarily spread by ticks and cause either Lyme borreliosis or relapsing fever. Substantial work has demonstrated the degree to which rodents and songbirds can contribute to the enzootic cycles and dispersal of these human diseases, but comparatively less attention has been paid to the role of wild bats, particularly in temperate regions. We here report human-relevant findings from a two-year, seasonal survey of migratory Mexican free-tailed bats (*Tadarida brasiliensis*) in Oklahoma, USA. We tested nearly 400 bats and identified *Borrelia puertoricensis*, a relapsing fever species that could infect humans. Importantly, this represents the first detection of *Borrelia puertoricensis* in bats and only the second detection in wild vertebrate hosts, expanding the known host range of this emerging tick-borne pathogen. Given the known migratory routes of Mexican free-tailed bats, our results have implications for the role that bats may play in tick-borne pathogen dispersal in North America.

## OBSERVATION

Bacteria in the genus *Borrelia* are primarily transmitted by ticks and cause two diseases of high human health concern: Lyme borreliosis (LB) and relapsing fever (RF). LB is the most common vector-borne disease in the Northern Hemisphere, with hard ticks (Ixodidae) serving as vectors of species in the *Borrelia burgdorferi sensu lato* complex (1). RF is globally distributed, with this clade of febrile illness–causing borreliae vectored by both hard and soft ticks (Argasidae) (2). Understanding the role that different host species play in maintaining enzootic cycles of borreliae and in dispersing these infections is important to ultimately predict human disease risk (3, 4).

While rodents and migratory birds are both understood to be important hosts in the epidemiology of LB and, to a lesser extent, RF borreliae (3–6), bats have received less attention for their role in the infection dynamics of these bacteria. However, bats can host RF borreliae that likely transmit to humans (7), and other borreliae hosted by bats in the tropics form novel clades adjacent to LB borreliae (8, 9). Despite this growing attention, the genetic diversity and zoonotic potential of bat-borne borreliae remains poorly understood, in part because the vast majority of work has focused on bats in the Neotropics, Afrotropics, or Australasian realm (10).

Fewer bat-borne *Borrelia* surveys have been conducted in temperate zones (11, 12), which could underestimate zoonotic potential and ignore a key aspect of *Borrelia* transmission. Most migratory bats occur in temperate zones, and physiological costs of migration in birds can cause chronic *Borrelia* infection to reactivate (13). Migration could thus allow bats to not only disperse ticks but also active *Borrelia* infections. Here, we assessed *Borrelia* diversity in the Mexican free-tailed bat (*Tadarida brasiliensis*), which undertakes migrations between wintering grounds in Mexico and large, summer roosts throughout the southwestern United States (14).

As part of a larger study of bat migration, immunity, and infection (15, 16), we collected blood from 386 Mexican free-tailed bats in western Oklahoma at monthly or biweekly intervals from March through October 2022 and 2023 (Fig. S1). We also opportunistically sampled pallid bats (*Antrozous pallidus*, *n* = 2), big brown bats (*Eptesicus fuscus*, *n* = 2), cave myotis (*Myotis velifer*, *n* = 11), and one hoary bat (*Lasiurus cinereus*). We captured bats with hand nets or mist nets and stored blood on Whatman FTA cards. Most of these 402 bats (75%) were inspected for ticks, with a subset collected and stored in 70% ethanol; we screened another four pallid bats, three Western big-eared bats (*Corynorhinus townsendii*), and 21 Mexican free-tailed bats for ticks but did not collect blood (*n* = 427). The spatial and temporal distribution of sampling is provided in Fig. S2. All bats were released at capture sites.

We used QIAamp DNA Investigator Kits (Qiagen) to extract DNA from bat blood (15). Where possible, we microscopically identified collected ticks (17) and extracted DNA using proteinase K digestion and *Quick*-DNA Miniprep Kits (Zymo). We confirmed tick taxonomy through PCR and Sanger sequencing of the *COI* gene (18). To determine *Borrelia* spp. infection, we used PCR and Sanger sequencing of the 16S rRNA and flagellin (*flaB*) genes (Table S1) (8). We aligned our sequences with references using MUSCLE and used MrBayes for phylogenetic analysis, with each gene tree run for 10,000,000 generations via a GTR +I + G model (19).

Across our two-year seasonal study, four bats were positive for borreliae (1%, 95% CI: 0.39%–2.53%), including three Mexican free-tailed bats and one pallid bat (Table S2). Only 6.14% of Mexican free-tailed bats were parasitized by ticks, while we found ticks on 33% and 30% of pallid bats and cave myotis, respectively. No other bat species hosted borreliae or ticks. For Mexican free-tailed bats, parasitized hosts had one (72%), two (24%), or three (4%) ticks. We confirmed ticks as argasids (e.g., see GenBank PX651872), but none of the three tested ticks were positive for borreliae. We then used generalized additive models to assess seasonal, annual, and sex variation in *Borrelia* infection and tick parasitism in Mexican free-tailed bats using cyclic smooth terms for week and the mgcv R package (20). Infection risk did not vary by week, year, or sex, while bat ticks were more likely in 2023 and after spring migration (Fig. S3; Table S3). Data on *Borrelia* and ticks have been deposited within the Pathogen Harmonized Observatory (21).

**Fig 1:**
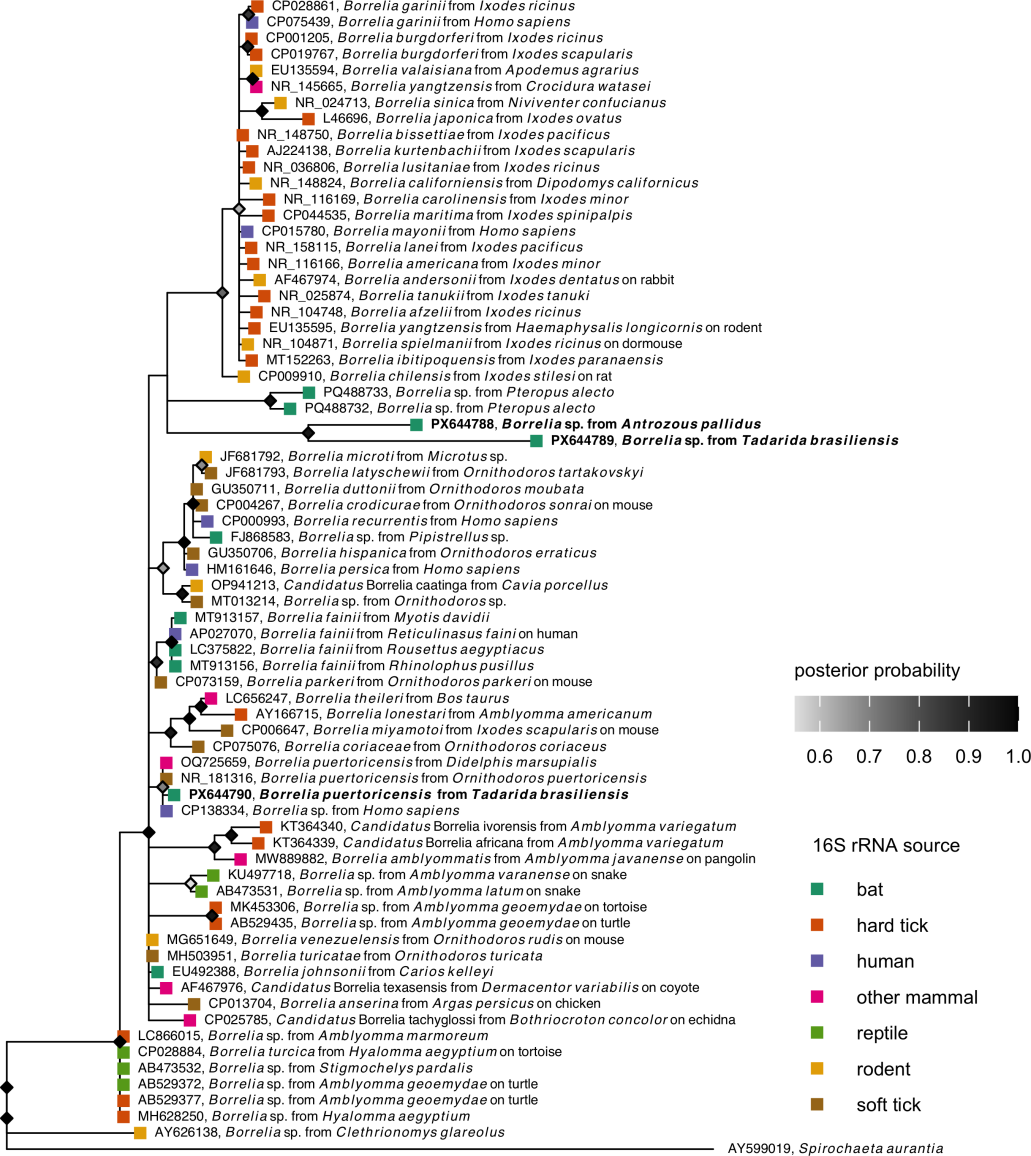
Consensus Bayesian phylogeny of the 16S rRNA *Borrelia* spp. sequences from this study (shown in bold) and reference sequences from bats, other mammals, reptiles, and ticks. Nodes are colored by posterior probability (nodes with less than 50% support are not shown).

We obtained 16S rRNA sequences from all positives, with *flaB* sequences from two of the Mexican free-tailed bats (Fig. 1). Two 16S rRNA sequences from Mexican free-tailed bats showed 99.9%–100% identity to *Borrelia puertoricensis* identified from soft ticks and opossums in the neotropics (22, 23). The *flaB* sequences were 97.7%–98.3% similar to *B. puertoricensis*, which confirms these bat borreliae within the RF clade (Fig. S4). Both positive bats (OK21 and OK535) were sampled just following or prior to migration (Fig. S3). 16S rRNA sequences from the other Mexican free-tailed bat and the pallid bat formed distinct lineages (92.2% identity). These two sequences were positioned near novel borreliae from *Pteropus* bats adjacent to the LB clade but shared only 89.3%–93.3% similarity with the pteropodid lineages (8). Sequences are available on GenBank through accession numbers PX644787–PX644790 (i.e., 16S rRNA) and PX644764 and PX644765 (i.e., *flaB*).

**Fig 2:**
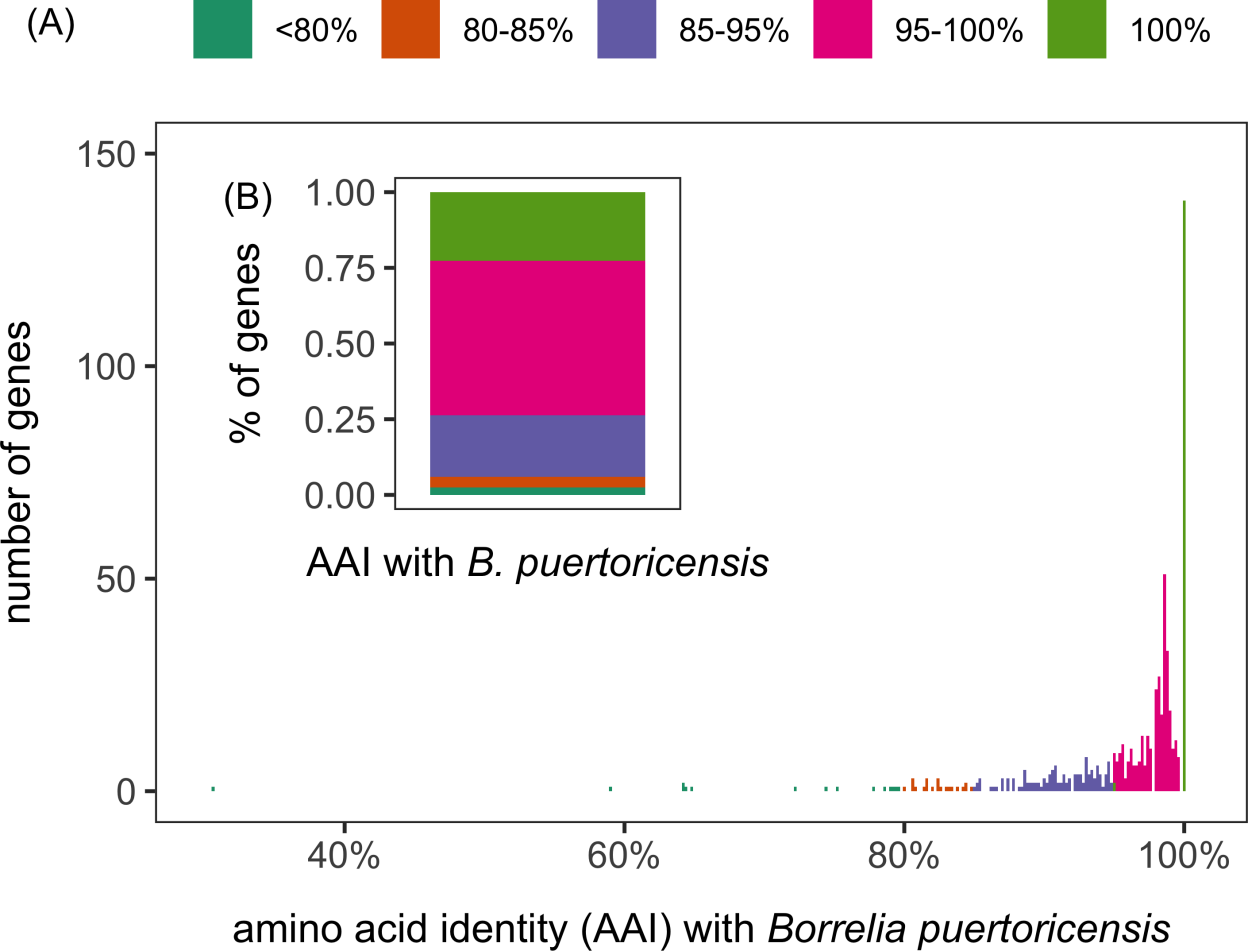
Amino acid identity (AAI) between the 622 *Borrelia* genes identified in the Mexican free-tailed bat sample (OK535) and those from *Borrelia puertoricensis*, using reference genomes from soft ticks (GCA_023035875 and NZ_CP149102) and a human (NZ_CP138334). Panel **A** shows the distribution of AAI, with inset **B** displaying the proportional composition of each AAI bin. See Tables S4 and S5 for raw AAI data and all *Borrelia* genomes used in the analyses.

Given the high similarity of these 16S rRNA and *flaB* sequences to *B. puertoricensis*, we attempted further characterization of the sample from bat OK535 using shotgun metagenomics (see the supplemental material). Given the low DNA volume, we first conducted whole-genome amplification using multiple displacement amplification via the TruePrime WGA Kit (Expedeon) (24). Illumina sequencing yielded 17.48 Gbp in 58.07 million 300-bp paired-end reads, available via accession numbers PRJNA1401797 (BioProject), SAMN54571938 (BioSample), and SRR36790398 (SRA). We identified 1,592 paired-end reads (0.003% of total reads) affiliated with the genus *Borrelia* (File S1). tBLASTx query of assembled contigs (File S2) identified 622 partial or complete protein-coding genes (Table S4). The absolute majority of genes displayed either identical (*n* = 139) or extremely high (>80%) amino acid identity (*n* = 467) to *B. puertoricensis* reference genomes from soft ticks and a human patient (Fig. 2; Table S5). These results corroborate *B. puertoricensis* as the only *Borrelia* species in the Mexican free-tailed bat sample.

We here demonstrate that migratory North American bats harbor distinct but LB-adjacent borreliae as well as RF infections. For the former, such results further support a novel clade of borreliae in bats spanning highly divergent host families (i.e., Pteropodidae, Molossidae, and Vespertilionidae) (8). Future work is needed to characterize the zoonotic potential of these bat-hosted lineages. For RF infections, we identified *B. puertoricensis* (22), representing the first detection of this lineage in bats and only the second detection in wild vertebrates (23). While these infections were rare, both detections were close to migratory periods. Further work is needed to understand the annual consistency of these seasonal patterns and whether migration could cause reactivation of chronic infections, as seen in birds (13). Tracking migratory origins is also needed to assess if such infections indicate dispersal from wintering grounds, given common tick infestations after spring migration and detection of *B. puertoricensis* in soft ticks in Mexico (25). Our findings thus warrant further attention to the role of migratory bats in the epidemiology and seasonal dispersal of RF borreliae as well as to the zoonotic risk of these infections.

## Data Availability

Sequences are available on GenBank through accession numbers PX644787–PX644790 (i.e., 16S rRNA) and PX644764 and PX644765 (i.e., flaB). Illumina sequencing reads are available via accession numbers PRJNA1401797 (BioProject), SAMN54571938 (BioSample), and SRR36790398 (SRA).

## SUPPLEMENTAL MATERIAL

File S1 msphere.00085-26-s0001.txt FASTA file of reads affiliated with the genus *Borrelia*.

File S2 msphere.00085-26-s0002.txt FASTA file of contigs assembled from *Borrelia* reads.

Supplemental Material msphere.00085-26-s0003.pdf Supplemental figures and tables.

Table S4 msphere.00085-26-s0004.xlsx BLAST results of all 622 partial or complete *Borrelia* genes identified.

Table S5 msphere.00085-26-s0005.xlsx Reference *Borrelia* genomes used in bioinformatic analyses.

ASM does not own the copyrights to Supplemental Material that may be linked to, or accessed through, an article. The authors have granted ASM a non-exclusive, world-wide license to publish the Supplemental Material files. Please contact the corresponding author directly for reuse.
